# An approach to localization for ensemble-based data assimilation

**DOI:** 10.1371/journal.pone.0191088

**Published:** 2018-01-19

**Authors:** Bin Wang, Juanjuan Liu, Li Liu, Shiming Xu, Wenyu Huang

**Affiliations:** 1 LASG, Institute of Atmospheric Physics, Beijing, China; 2 Ministry of Education Key Laboratory for Earth System Modeling, Department of Earth System Science, Tsinghua University, Beijing, China; 3 University of Chinese Academy of Sciences, Beijing, China; Universidade de Aveiro, PORTUGAL

## Abstract

Localization techniques are commonly used in ensemble-based data assimilation (e.g., the Ensemble Kalman Filter (EnKF) method) because of insufficient ensemble samples. They can effectively ameliorate the spurious long-range correlations between the background and observations. However, localization is very expensive when the problem to be solved is of high dimension (say 10^6^ or higher) for assimilating observations simultaneously. To reduce the cost of localization for high-dimension problems, an approach is proposed in this paper, which approximately expands the correlation function of the localization matrix using a limited number of principal eigenvectors so that the Schür product between the localization matrix and a high-dimension covariance matrix is reduced to the sum of a series of Schür products between two simple vectors. These eigenvectors are actually the sine functions with different periods and phases. Numerical experiments show that when the number of principal eigenvectors used reaches 20, the approximate expansion of the correlation function is very close to the exact one in the one-dimensional (1D) and two-dimensional (2D) cases. The new approach is then applied to localization in the EnKF method, and its performance is evaluated in assimilation-cycle experiments with the Lorenz-96 model and single assimilation experiments using a barotropic shallow water model. The results suggest that the approach is feasible in providing comparable assimilation analysis with far less cost.

## Introduction

The statistical accuracy of background error is extremely important for any data assimilation scheme, and the background error covariance matrix (the **B** matrix, hereinafter) is often estimated from ensembles[1–4]. However, for practical applications in the ocean or atmosphere, current computational resources limit the ensemble size, which is often much smaller than both the dimension of the model and the number of observations. A small ensemble size is very likely to introduce sampling errors, leading to (a) underestimation of ensemble spread and (b) spurious correlations over long distances or between variables known to be uncorrelated [5]. Common approaches to reduce sampling errors in ensemble-based data assimilation (EDA) include covariance inflation[6–10] and localization[2,3,9,11–26].

In covariance inflation algorithms, the prior ensemble state covariance is increased by linearly inflating each scalar component of the state vector before assimilating observations [5,9,27]. By reducing the underestimation of the **B** matrix, covariance inflation plays an important role in preventing filter divergence of EDA. In addition, a proper localization of the estimated **B** matrix is needed to reduce spurious non-zero long-range correlations in the **B** matrix and to improve its rank deficiency, which allow ensemble-based assimilation schemes with an ensemble size fewer than 100 members to work properly with realistic atmosphere and ocean models [9,25].

Localization is usually implemented as a Schür product between the ensemble-based **B** matrix and a correlation matrix of which the elements are calculated according to a correlation function with respect to their coordinates. A commonly-used correlation function is the Quasi-Gaussian compactly supported form proposed by Gaspari and Cohn [28] (referred to simply as the GC localization or the correlation function, hereinafter). However, given that the optimal localization is likely to depend on the ensemble configuration (e.g., ensemble size, observation types), a comprehensive tuning of localization is needed in practice. In order to avoid the challenge of tuning the localization parameters [5, 25], adaptive localization functions have been proposed [9,17]. As noted in Fertig et al. [19], spatial localization is still difficult when assimilating satellite observations. To tackle this problem, Fertig et al. [19] updated the state at a given location through assimilating satellite observations that are strongly correlated to the model state there. In addition, Miyoshi and Sato [29] and Campbell et al.[30] explored localization functions for satellite radiances. In Miyoshi and Sato [29], the normalized sensitivity function of satellite sensors was used as the localization weights, whereas in Campbell et al.[30] forward operators performing weighted averages of a large number of state variables were applied. Zhu et al. [24] also proposed a localization scheme to use non-local observations; their basic idea is similar to that of Liu et al. [21].

Despite the differences among various localization approaches, the computational cost of localization algorithms is always an important issue as more and more observations are used. In the following, we provide a simple comparison between the computational costs with and without localization, based on the ensemble Kalman Filter (EnKF) with simultaneous treatment for assimilating observations.

Let *m*_*x*_, *m*_*y*_ and *n* be the number of control variables, the number of observations and the ensemble size, respectively. In the EnKF approach, the forecast error covariance matrix **P**^*f*^ is calculated as follows:

$$\left\{ \begin{array}{l} P^{f}\approx bb^{T}\\ b=b_{m_{x}\times n}=\frac{1}{\sqrt{n-1}}(x_{1}-\bar{x},x_{2}-\bar{x},\cdots ,x_{n}-\bar{x})\\ \bar{x}=\frac{1}{n}(x_{1}+x_{2}+\cdots +x_{n}) \end{array}.\right.$$ (1)

The gain matrix is then:

$$K_{\left\{full\right\}}=P^{f}H^{T}{\left({HP}^{f}H^{T}+R\right)}^{-1},$$ (2)

where **H** is the observation operator (*m*_*y*_ × *m*_*x*_ matrix) and **R** is the observational error covariance (*m*_*y*_ × *m*_*y*_ matrix). Since *n* is usually smaller than *m*_*x*_, the sample covariance matrix is rank deficient. A Schür product is then implemented between the covariance matrix and a correlation matrix to increase the rank through removing spurious long-range correlations. Then, the Kalman gain is written as

$$K_{\left\{loc\right\}}=(\rho_{m_{x}\times m_{x}}\circ P^{f})H^{T}\left(H(\rho_{m_{x}\times m_{x}}\circ P^{f}\right)H^{T}+R{)}^{-1},$$ (3)

where $\rho_{m_{x}\times m_{x}}$ is a compactly supported correlation matrix in which each column represents spatial correlations at a given model gridpoint. This method is referred to as model spatial localization. Following Houtekamer and Mitchell [13], localization has also been applied in observation space, leading to the following Kalman gain:

$$K_{\left\{loc\right\}}=\rho_{m_{x}\times m_{y}}\circ (P^{f}H^{T})\left(\rho_{m_{y}\times m_{y}}\circ (HP^{f}H^{T}\right)+R{)}^{-1}.$$ (4)

Since

$$Hb\approx P_{m_{y}\times n}^{y}=\frac{1}{\sqrt{n-1}}(y_{1}-\bar{y},y_{2}-\bar{y},\cdots ,y_{n}-\bar{y});\bar{y}=\frac{1}{n}\sum_{i=1}^{n}y_{i},$$ (5)

where **P**^y^ is a *m*_*y*_ × *n* matrix, Eqs (2) and (4) can be respectively expressed as

$$K_{\left\{full\right\}}=K_{m_{x}\times m_{y}}=b_{m_{x}\times n}{\left(P_{m_{y}\times n}^{y}\right)}^{T}{\left({P_{m_{y}\times n}^{y}(P_{m_{y}\times n}^{y})}^{T}+R_{m_{y}\times m_{y}}\right)}^{-1},$$ (6)

and

$$K_{\left\{loc\right\}}=K_{m_{x}\times m_{y}}=\rho_{m_{x}\times m_{y}}\circ [b_{m_{x}\times n}{\left(P_{m_{y}\times n}^{y}\right)}^{T}]{\left({{\rho_{m_{y}\times m_{y}}\circ [P}_{m_{y}\times n}^{y}(P_{m_{y}\times n}^{y})}^{T}]+R_{m_{y}\times m_{y}}\right)}^{-1}.$$ (7)

When the initial analysis increment ${x_{m_{x}}^{\prime }=K}_{m_{x}\times m_{y}}y_{m_{y}}^{\prime obs}$ is calculated using Eqs (6) and (7), respectively, their costs are quite different, where $y_{m_{y}}^{\prime obs}$ is the *m*_*y*_-dimension observation innovation vector. If we ignore the difference between the costs for calculating: $y_{m_{y}}^{r}={\left({P_{m_{y}\times n}^{y}(P_{m_{y}\times n}^{y})}^{T}+R_{m_{y}\times m_{y}}\right)}^{-1}y_{m_{y}}^{\prime obs}$ and $y_{m_{y}}^{rl}={\left(\rho_{m_{y}\times m_{y}}\circ [{P_{m_{y}\times n}^{y}(P_{m_{y}\times n}^{y})}^{T}]+R_{m_{y}\times m_{y}}\right)}^{-1}y_{m_{y}}^{\prime obs}$, which are relatively small, the calculation of the increment with localization using Eq (7) (i.e., $x_{m_{x}}^{\prime }=\rho_{m_{x}\times m_{y}}\circ [b_{m_{x}\times n}{\left(P_{m_{y}\times n}^{y}\right)}^{T}]y_{m_{y}}^{rl}$) needs *m*_*x*_ × *m*_*y*_ × (*n +* 2) multiplications and *m*_*x*_ × *m*_*y*_ × *n − m*_*x*_ additions, while that without localization using Eq (6) (i.e., $x_{m_{x}}^{\prime }=[b_{m_{x}\times n}{\left(P_{m_{y}\times n}^{y}\right)}^{T}]y_{m_{y}}^{r}$) takes only (*m*_*x*_ + *m*_*y*_) × *n* multiplications and (*m*_*x*_ + *m*_*y*_ *−* 1) × *n − m*_*x*_ additions.

To provide an intuitive understanding of the huge cost of localization in the EnKF when assimilating multi-source observations, including satellite measurements, let us consider a typical realistic NWP configuration such that *m*_*x*_ = 10^7^, *m*_*y*_ = 10^5^ and *n* = 30. In this case, the calculation of the increment with localization takes about 3 × 10^13^ multiplications and about 3 × 10^13^ additions, much more expensive than without localization, which takes about 3 × 10^8^ multiplications and 3 × 10^8^ additions.

To reduce the huge cost of localization in the EnKF, two kinds of methods are frequently used, including batch processing [2] and serial processing [14]. In batch processing, the observations are organized into batches and each batch is assimilated simultaneously, while in serial processing, observations are assimilated sequentially, one by one. However, even if batch or serial processing is performed in the EnKF, the computational cost is still quite large in practice. For example, the EnKF with serial processing needs *m*_*y*_ sequential assimilations and, each time at least *m*_*x*_ × *n* multiplications are required according to the gain matrix formula (see Eq (6)). In total, at least *m*_*x*_ × *m*_*y*_ ×*n* multiplications are needed for the serial implementation of the EnKF. This is still a huge cost. As in the previous example, let *m*_*x*_, *m*_*y*_ and *n* be 10^7^, 10^5^ and 30, respectively; the EnKF will perform at least 3×10^13^ multiplications. Obviously, the cost reduction by serial processing is not significant. The cost of batch processing can be estimated in the same way, and is just as large as the serial processing. The obvious advantage of these two methods is easier to obtain the solutions to $y_{m_{y}}^{rl}={\left(\rho_{m_{y}\times m_{y}}\circ {P_{m_{y}\times n}^{y}(P_{m_{y}\times n}^{y})}^{T}+R_{m_{y}\times m_{y}}\right)}^{-1}y_{m_{y}}^{\prime obs}$, which is much less costly than that of $x_{m_{x}}^{\prime }=\rho_{m_{x}\times m_{y}}\circ [b_{m_{x}\times n}{\left(P_{m_{y}\times n}^{y}\right)}^{T}]y_{m_{y}}^{rl}$.

In this paper, we proceed as follows: first a more detail descriptions of the new scheme are given. Then, we compare its filtering performance with the standard scheme. We also conduct preliminary tests using the new approach in the EnKF. Finally, we discuss our methods, the scope and limitations of this study, and some of the possible extension.

## Materials and methods

### Methodology

The basic idea of covariance localization is to limit the number of observations that can affect the analysis at a particular gridpoint. A simple technique for this is through observation selection, since the analysis is affected by observations within a cutoff radius [2]. Another way to implement covariance localization is to apply a Schür product between the forecast error covariance matrix and a correlation matrix [13], either in model space (Eq (3)) or in observation space (Eq (4)). For example, the element ρ_*i*,*j*_ of the correlation matrix $\rho_{m_{x}\times m_{y}}$ can be written as

$$\rho_{i,j}=C_{0}(\frac{d_{i,j}^{h}}{d_{0}^{h}})∙C_{0}(\frac{d_{i,j}^{v}}{d_{0}^{v}}),(i=1,2,\cdots ,m_{x};j=1,2,\cdots ,m_{y}),$$ (8)

where $d_{0}^{h}$ and $d_{0}^{v}$ are the prescribed horizontal and vertical filtering radii, respectively; $d_{i,j}^{h}$ and $d_{i,j}^{v}$ are the horizontal and vertical distances between the *i*-th control variable and the *j*-th observation, respectively. *C*_0_ in (8) is the GC correlation function:

$$C_{0}(r)=C_{0}(L_{i},L_{j})=\left\{ \begin{array}{l} -\frac{1}{4}r^{5}+\frac{1}{2}r^{4}+\frac{5}{8}r^{3}-\frac{5}{3}r^{2}+1,\;0\leq r\leq 1\\ \frac{1}{12}r^{5}-\frac{1}{2}r^{4}+\frac{5}{8}r^{3}+\frac{5}{3}r^{2}-5r+4-\frac{2}{3}r^{-1},1<r\leq 2\\ 0,\;2<r \end{array}\right.$$ (9)

where $=\frac{d_{i,j}}{d_{0}}$, *L*_*i*_ and *L*_*j*_ are the spatial coordinates of the *i*-th control variable and the *j*-th observation, respectively. From Eqs (6) and (7), it is clear that the increase in computational cost due to the localization is mainly caused by the Schür product between $b_{m_{x}\times n}{\left(P_{m_{y}\times n}^{y}\right)}^{T}$ and the correlation matrix $\rho_{m_{x}\times m_{y}}$, leading to the change of $b_{m_{x}\times n}{\left(p_{m_{y}\times n}^{y}\right)}^{T}$ from a separable form to an inseparable form. This expensive calculation can fully be avoided and can be reduced to a time-saving product between $b_{m_{x}\times n}$ and an *n*-dimensional vector if the Schür product is not performed.

If the localization matrix $\rho_{m_{x}\times m_{y}}$ can possibly be decomposed into a product of two vectors:

$$\rho_{m_{x}\times m_{y}}=\rho_{m_{x}}^{x}{\left(\rho_{m_{y}}^{y}\right)}^{T},$$ (10)

the aforementioned Schür product may become separable:

$$\rho_{m_{x}\times m_{y}}\circ [b_{m_{x}\times n}{\left(P_{m_{y}\times n}^{y}\right)}^{T}]={\tilde{b}}_{m_{x}\times n}{\left({\tilde{P}}_{m_{y}\times n}^{y}\right)}^{T},$$ (11)

where $\rho_{m_{x}}^{x}$ and $\rho_{m_{y}}^{y}$ are *m*_*x*_- and *m*_*y*_-dimension vectors, respectively, and ${\tilde{b}}_{m_{x}\times n}$ and ${\tilde{P}}_{m_{y}\times n}^{y}$ are:

$$\{\begin{array}{c} {\tilde{b}}_{m_{x}\times n}=\frac{1}{\sqrt{n-1}}(\rho_{m_{x}}^{x}\circ (x_{1}-\bar{x}),\rho_{m_{x}}^{x}\circ (x_{2}-\bar{x}),\cdots ,\rho_{m_{x}}^{x}\circ (x_{n}-\bar{x}))\\ {\tilde{p}}_{m_{y}\times n}^{y}=\frac{1}{\sqrt{n-1}}(\rho_{m_{y}}^{y}\circ (y_{1}-\bar{y}),\rho_{m_{y}}^{y}\circ (y_{2}-\bar{y}),\cdots ,\rho_{m_{y}}^{y}\circ (y_{n}-\bar{y})) \end{array}.$$ (12)

In this way, the high computational cost resulting from the localization can be greatly reduced.

However, this localization matrix cannot be expressed in the form of Eq (10), because it is impossible to decompose the correlation function into the following form:

$$C_{0}(r)=C_{0}(L_{i},L_{j})=C_{0}^{x}(L_{i})∙C_{0}^{y}(L_{j}),$$ (13)

according to its definition by Eq (9). How to decompose the correlation function becomes the key point to reduce high computational cost for localization. Liu et al. [21] used the empirical orthogonal function (EOF) to decompose the correlation function on a low-resolution grid, and then interpolated the chosen dominant modes to the high-resolution grid. This is one of the earliest studies to expand the GC localization function. It is an efficient method that avoids the high cost of conducting the EOF on the high-resolution model grid directly, but it inevitably results in a reduction of accuracy in calculating the correlation function, due to the low precision of the leading modes decomposed on the low-resolution grid and the interpolation from the low-resolution grid to the high-resolution grid. Buehner et al. [31–32], Bishop et al. [33] and Kuhl et al. [34] adopted scaled spherical harmonics to decompose the correlation function. They provided an analytical and continuous expansion of the GC localization function. However, it is difficult to apply these methods to regional assimilations, because the spherical harmonics that requires homogeneous or periodic boundary conditions is more suitable for a spherical domain. Actually, due to the same reason, regional models rarely use the spherical harmonics for discretization of their dynamical cores. To avoid the aforesaid problems, this study tries to find a group of basis functions to expand the correlation function:

$$C_{0}(r)=C_{0}(L_{i},L_{j})=\overset{K_{c}}{\underset{k=1}{\sum }}\beta_{k}e_{k}(L_{i})e_{k}(L_{j}),$$ (14)

so that the expansion is applicable for assimilations in both spherical and rectangular domains. The basis function *e*_*k*_(**L**) in (14) is analytical and subject to orthogonality as follows:

$$\int_{\Omega }w(L)e_{k}(L)e_{l}(L)ds=\{\begin{array}{cc} 1 & \mathrm{if}k=l\\ 0 & \mathrm{if}k\neq l \end{array},$$ (15)

where *w*(*x*) is a weighting function and Ω is the domain of the model. The coefficient *β*_*k*_, which is the eigenvalue (or variance) of the *k*-th basis function, can be calculated directly according to the above orthogonality (Eq (15)):

$$\beta_{k}=\int_{\Omega }\int_{\Omega }w(L_{1})w(L_{2})C_{0}(L_{1},L_{2})e_{k}(L_{1})e_{k}(L_{2})ds_{1}ds_{2}.$$ (16)

*K*_*c*_ in (14) is the number of basis functions, which is either infinite when *β*_*k*_ is calculated based on Eq (16), or a finite positive integer depending on the given resolution to discretely calculate the coefficient. If the orthogonal basis functions are just the intrinsic modes of the correlation function, a finite number of leading modes can be chosen to express the correlation function approximately as follows:

$$C_{0}(r)=C_{0}(L_{i},L_{j})\approx C_{K_{0}}(L_{i},L_{j})=\overset{K_{0}}{\underset{k=1}{\sum }}\beta_{k}e_{k}(L_{i})e_{k}(L_{j}),$$ (17)

where *K*_0_ is the number of selected leading modes, which should be much smaller than *K*_*c*_. *K*_0_ can be determined according to a given criterion for the contribution of accumulated variance of the chosen leading modes to the total variance: $\sum_{k=1}^{K_{0}}\beta_{k}/\sum_{k=1}^{K_{c}}\beta_{k}$ (say, 95% or more). In this way, the localization matrix $\rho_{m_{x}\times m_{y}}$ can be simplified to the following form:

$$\rho_{m_{x}\times m_{y}}\approx \overset{K_{0}}{\underset{k=1}{\sum }}\rho_{m_{x}}^{\left(x,k\right)}{\left(\rho_{m_{y}}^{\left(y,k\right)}\right)}^{T}.$$ (18)

In Eq (18), $\rho_{m_{x}}^{\left(x,k\right)}$ and $\rho_{m_{y}}^{\left(y,k\right)}$ are *m*_*x*_-dimension and *m*_*y*_-dimension vectors, respectively. Now, the localization can be reduced into

$$\left\{ \begin{array}{c} \rho_{m_{x}\times m_{y}}\circ [b_{m_{x}\times n}{\left(P_{m_{y}\times n}^{y}\right)}^{T}]\approx \sum_{k=1}^{K_{0}}b_{m_{x}\times n}^{\left(k\right)}{\left(P_{m_{y}\times n}^{\left(k\right)}\right)}^{T}\\ {{\rho_{m_{y}\times m_{y}}\circ [P}_{m_{y}\times n}^{y}(P_{m_{y}\times n}^{y})}^{T}]\approx \sum_{k=1}^{K_{0}}P_{m_{y}\times n}^{\left(k\right)}{\left(P_{m_{y}\times n}^{\left(k\right)}\right)}^{T} \end{array},\right.$$ (19)

where

$$\{\begin{array}{c} b_{m_{x}\times n}^{\left(k\right)}=\frac{1}{\sqrt{n-1}}(\rho_{m_{x}}^{\left(x,k\right)}\circ (x_{1}-\bar{x}),\rho_{m_{x}}^{\left(x,k\right)}\circ (x_{2}-\bar{x}),\cdots ,\rho_{m_{x}}^{\left(x,k\right)}\circ (x_{n}-\bar{x}))\\ p_{m_{y}\times n}^{\left(k\right)}=\frac{1}{\sqrt{n-1}}(\rho_{m_{y}}^{\left(y,k\right)}\circ (y_{1}-\bar{y}),\rho_{m_{y}}^{\left(y,k\right)}\circ (y_{2}-\bar{y}),\cdots ,\rho_{m_{y}}^{\left(y,k\right)}\circ (y_{n}-\bar{y})) \end{array}.$$ (20)

The new localization scheme mainly needs (*m*_*x*_ + *m*_*y*_) × *n* × *K*_0_ × 2 multiplications and ((*m*_*x*_ + *m*_*y*_ *−* 1) × *n − m*_*x*_) × *K*_0_ additions. Under the same resolutions as mentioned in the introduction (*m*_*x*_ = 10^7^, *m*_*y*_ = 10^5^, and *n* = 30), and if *K*_0_ = 20, the multiplication and addition calculations are performed about 1.2×10^10^ and 6×10^9^ times, respectively, in the new scheme. Its cost is roughly 2500 times lower than usual. In the next subsection, we will give the definition of the basis functions, and then investigate the precision of the expansion in the right side of Eq (17) in 1D and 2D cases.

### Basis functions

As discussed above, expanding the correlation function by means of a group of basis functions can greatly reduce the cost of the localization. Therefore, the construction of basis functions is the first step for the expansion. Actually, the eigenvectors of the correlation function on the discrete grid with a prescribed resolution can be used to determine the features of the basis functions and, ultimately, to construct them analytically. For this purpose, the eigenvectors of the discrete correlation function are investigated first in the following. For convenience of discussion, investigations are conducted in the 1D case under periodic and non-periodic boundaries, respectively.

#### 1D case under non-periodic boundary condition

Suppose the domain of definition is [*a*, *b*], of which the length is *l*_0_ = *b* − *a*. Uniformly partition the interval [*a*, *b*] using *m* grids whose locations are *x*_*i*_ = *a*+(*i*−1)×*dx*, where *dx* = *l*_0_/(*m*−1); *i* = 1,2,⋯, *m*. For any *x*_*i*_ and *x*_*j*_ in [*a*, *b*], their distance is defined as *d*_*i*,*j*_ = |*x*_*i*_−*x*_*j*_|. If the filtering radius is *d*_0_, the non-dimensional distance can be expressed as *r*_*i*,*j*_ = *d*_*i*,*j*_/*d*_0_ = |*x*_*i*_−*x*_*j*_|/*d*_0_. In this way, the value of the correlation function between the grids can be calculated according to Eq (2): *c*_*i*,*j*_ = *C*_0_(*r*_*i*,*j*_) (*i* = 1,2,⋯, *m*; *j* = 1,2,⋯, *m*), which, as the elements, forms the localization matrix $\rho_{{}^{m\times m}}^{non-periodic}$, a sparse banded matrix. Because this matrix is symmetric, it has *m* real non-negative eigenvalues *σ*_1_ ≥ *σ*_2_ ≥ ⋯ *σ*_*m*_ ≥ 0 and corresponding unit orthogonal eigenvectors **s**_1_, **s**_2_,⋯, **s**_*m*_, so that

$$\rho_{m\times m}^{non-periodic}=\left[\begin{array}{cccc} c_{11} & c_{12} & \cdots & c_{1m}\\ c_{21} & c_{22} & \cdots & c_{2m}\\ \vdots & \vdots & \ddots & \vdots \\ c_{m1} & c_{m2} & \cdots & c_{mm} \end{array}\right]=\left(s_{1},s_{2},\cdots ,s_{m}\right)\left[\begin{array}{cccc} \sigma_{1} & 0 & \cdots & 0\\ 0 & \sigma_{2} & \cdots & 0\\ \vdots & \vdots & \ddots & \vdots \\ 0 & 0 & \cdots & \sigma_{m} \end{array}\right]\left[\begin{array}{c} s_{1}^{T}\\ s_{2}^{T}\\ \vdots \\ s_{m}^{T} \end{array}\right]=\overset{m}{\underset{k=1}{\sum }}\sigma_{k}s_{k}s_{k}^{T}.$$ (21)

When *m* is very large, *K*_0_ leading eigenvectors with the largest eigenvalues can be chosen to approximately expand the localization matrix:

$$\rho_{{}^{m\times m}}^{non-periodic}\approx \overset{K_{0}}{\underset{k=1}{\sum }}\sigma_{k}s_{k}s_{k}^{T}.$$ (22)

This means that the eigenvectors of the localization matrix can be the best choice for the basis functions in the discrete case. Therefore, we are interested in what analytical forms they have.

**Fig 1:**
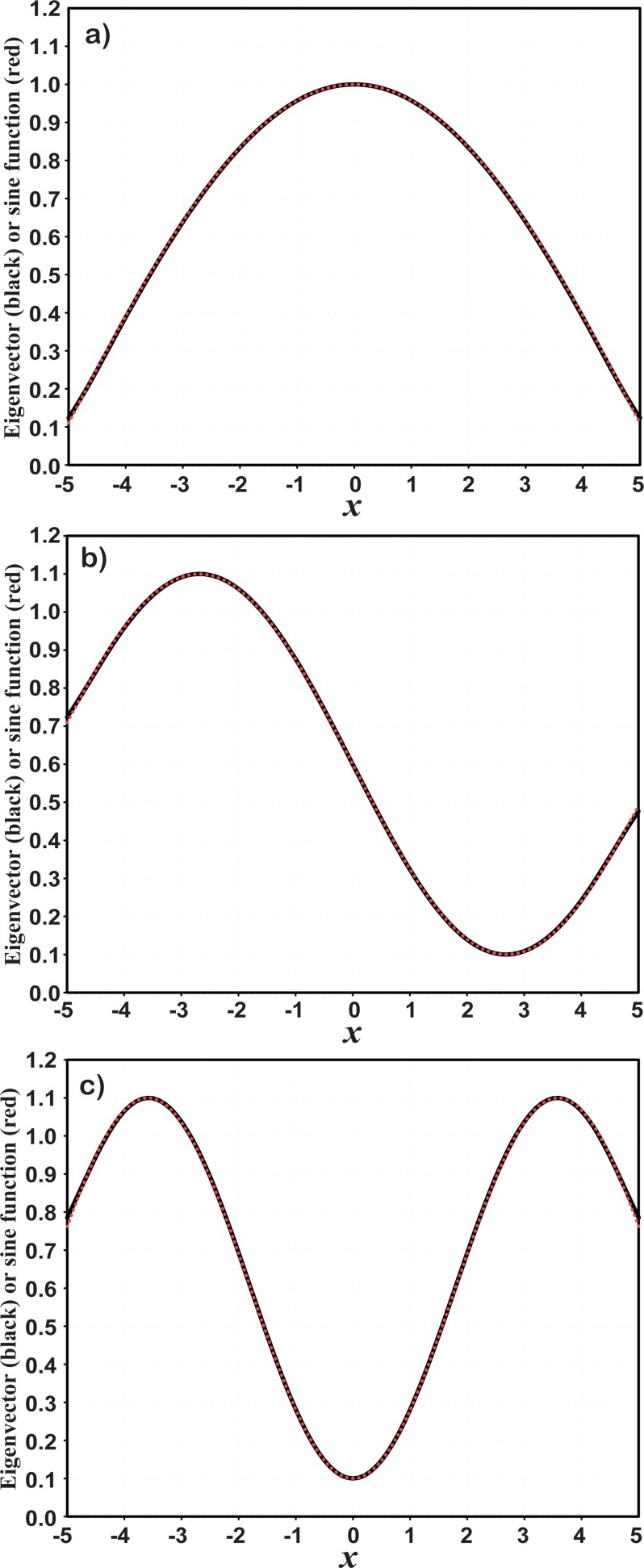
Leading eigenvectors of the correlation function (black solid) and sine function $\sin\frac{k\pi }{l}(x-\tilde{a})$ (red dotted), including a) the first eigenvector and sine function with *k* = 1, b) the second eigenvector and sine function with *k* = 2, and c) the third eigenvector and sine function with *k* = 3. Note that the two curves overlap in each panel.

**Fig 2:**
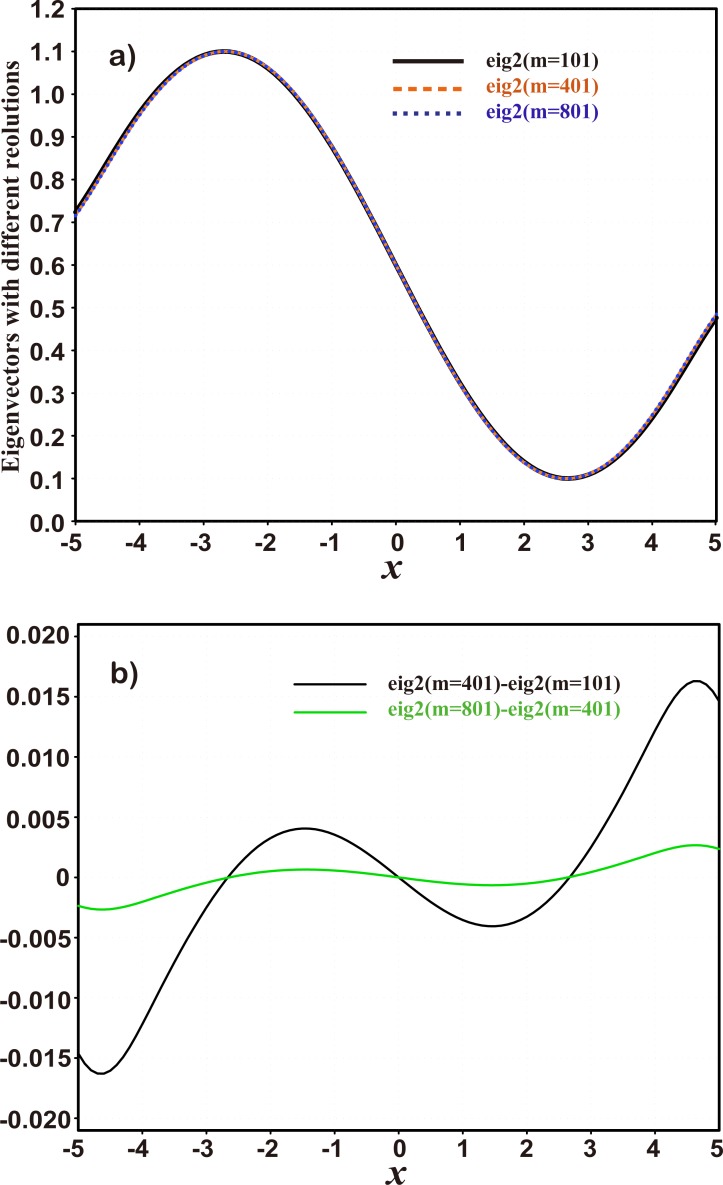
a) The second eigenvectors at different spatial resolutions including *m* = 101 (black solid), *m* = 401 (orange yellow dashed) and *m* = 801 (blue dotted); and b) the differences of eigenvectors between the resolutions of m = 101 and m = 401 (black line) and between the resolutions of m = 401 and m = 801.

Set *a* = −5.0, *b* = 5.0, *d*_0_ = 1.0, and *m* = 101. The eigenvectors can be easily calculated under this resolution, and their spatial distributions can also be depicted. For example, the spatial distributions of the first three eigenvectors (black solid lines) are shown in Fig 1. They are very close to the sine waves (see red dotted lines) $\sin\frac{k\pi }{l}(x_{i}-\tilde{a})$ (*k* = 1, 2, 3; *i* = 1, 2,⋯, *m*) that are incomplete in the domain of definition and are defined on an extended domain $\left[\tilde{a},\tilde{b}\right]$, where $\tilde{a}=a-\frac{l-l_{0}}{2},\tilde{b}=b+\frac{l-l_{0}}{2}$, and *l* > *l*_0_, because the values at the beginning and ending points of the interval [*a*, *b*] are not zero. Furthermore, when the resolution is increased, the wave shapes of the eigenvectors change very little. Fig 2A shows the second eigenvectors as an example with different resolutions of *m* = 101 (black solid line), *m* = 401 (brown dashed line) and *m* = 801 (blue dotted line), respectively, which barely differ from each other. Their differences between two adjacent resolutions are much smaller than the eigenvectors themselves in terms of amplitude (Fig 2B). In particular, these differences become smaller as resolution increases (Fig 2B). This suggests that sine-function-based eigenvectors are insensitive to grid resolution. On the other hand, the relative change of the extended boundary, defined as $\varepsilon =\frac{l-l_{0}}{l_{0}}$, is in a small range [0.066, 0.075] when the resolution varies from *m* = 101 to *m* = 801. It indicates that the analytical forms of the eigenvectors can be sine functions with different frequencies approximately so that they can be used as the basis functions:

$$e_{k}(x)=\sin\frac{k\pi }{l}(x-\tilde{a})\;(l=\tilde{b}-\tilde{a}=(1+\varepsilon )l_{0};\;k=1,2,\cdots ,K_{0}).$$ (23)

These functions are orthogonal on the extended domain of definition $\left[\tilde{a},\tilde{b}\right]$:

$$\int_{\tilde{a}}^{\tilde{b}}w(x)\sin\frac{k_{1}\pi }{l}(x-\tilde{a})\sin\frac{k_{2}\pi }{l}(x-\tilde{a})dx=\{\begin{array}{c} 1,\;\mathrm{if}k_{1}=k_{2}\\ 0,\;\mathrm{if}k_{1}\neq k_{2} \end{array},$$ (24)

where $w(x)=\frac{2}{l}$. Using the above sine functions, the correlation function can be approximately expressed by a truncated expansion:

$$C_{0}(r)=C_{0}(x_{1},x_{2})\approx C_{K_{0}}(x_{1},x_{2})=\overset{K_{0}}{\underset{k=1}{\sum }}\beta_{k}\sin\frac{k\pi }{l}(x_{1}-\tilde{a})\sin\frac{k\pi }{l}(x_{2}-\tilde{a}),$$ (25)

where

$$\beta_{k}=\frac{4}{l^{2}}\int_{\tilde{a}}^{\tilde{b}}\int_{\tilde{a}}^{\tilde{b}}C_{0}(x_{1},x_{2})\sin\frac{k\pi }{l}(x_{1}-\tilde{a})\sin\frac{k\pi }{l}(x_{2}-\tilde{a})dx_{1}dx_{2}.$$ (26)

#### 1D case under periodic boundary condition

In this case, the domain of definition is supposed to be a zonal circle at any a latitude *θ*, i.e., the longitude *λ* varies from *a* = 0 to *b* = 2*π*. Uniformly partition the interval [*a*, *b*] using *m* grids whose locations are *λ*_*i*_ = (*i*−1)×*dλ*, where *dλ* = 2*π*/(*m*−1); *i* = 1,2,⋯, *m*. For any *λ*_*i*_ and *λ*_*j*_ on the zonal circle, their distance can be defined as their arc length: *d*_*i*,*j*_ = *R*_0_ cos*θ*×min(|*λ*_*i*_−*λ*_*j*_|,2*π*−|*λ*_*i*_−*λ*_*j*_|) because of the periodic boundary, where *R*_0_ is the radius of the Earth. When the filtering radius of longitude is *λ*_0_, the geometrical filtering radius is then *d*_0_ = *R*_0_ cos*θ*×*λ*_0_. Consequently, the non-dimensional distance between *λ*_*i*_ and *λ*_*j*_ is *r*_*i*,*j*_ = *d*_*i*,*j*_/*d*_0_ = min(|*λ*_*i*_−*λ*_*j*_|,2*π*−|*λ*_*i*_−*λ*_*j*_|)/*λ*_0_, with which the localization matrix $\rho_{m\times m}^{periodic}$ calculated according to Eq (9) is still a symmetric matrix, but not a banded matrix. Similarly, it has *m* real non-negative eigenvalues *σ*_1_ ≥ *σ*_2_ ≥ ⋯ ≥*σ*_*m*_ ≥ 0 and corresponding unit orthogonal eigenvectors **s**_1_, **s**_2_,⋯, **s**_*m*_, so that

$$\rho_{m\times m}^{periodic}=\left[\begin{array}{cccc} c_{11} & c_{12} & \cdots & c_{1m}\\ c_{21} & c_{22} & \cdots & c_{2m}\\ \vdots & \vdots & \ddots & \vdots \\ c_{m1} & c_{m2} & \cdots & c_{mm} \end{array}\right]=\left(s_{1},s_{2},\cdots ,s_{m}\right)\left[\begin{array}{cccc} \sigma_{1} & 0 & \cdots & 0\\ 0 & \sigma_{2} & \cdots & 0\\ \vdots & \vdots & \ddots & \vdots \\ 0 & 0 & \cdots & \sigma_{m} \end{array}\right]\left[\begin{array}{c} s_{1}^{T}\\ s_{2}^{T}\\ \vdots \\ s_{m}^{T} \end{array}\right]=\overset{m}{\underset{k=1}{\sum }}\sigma_{k}s_{k}s_{k}^{T}$$ (27)

As decomposed above, the spatial distributions of the eigenvectors can also approximately be expressed as sine functions with different frequencies and phases. They can be defined with a periodic domain [*a*, *b*] as:

$$e_{k}(\lambda )=\sin\left(\frac{k\pi }{l}(\lambda -a)+\omega_{k}\right)\;(l=b-a;\;k=1,2,\cdots ,K_{0}).$$ (28)

These functions are orthogonal in the domain of definition [*a*,*b*]:

$$\int_{a}^{b}w(\lambda )\sin\left(\frac{k_{1}\pi }{l}(\lambda -a)+\omega_{k_{1}}\right)\sin\left(\frac{k_{2}\pi }{l}(\lambda -a)+\omega_{k_{2}}\right)d\lambda =\{\begin{array}{c} 1,\;\mathrm{if}k_{1}=k_{2}\\ 0,\;\mathrm{if}k_{1}\neq k_{2} \end{array},$$ (29)

where $w(\lambda )=\frac{2}{l}$. Using the above sine functions, the correlation function can be approximately expressed by a truncated expansion:

$$C_{0}(r)=C_{0}(\lambda_{1},\lambda_{2})\approx C_{K_{0}}(\lambda_{1},\lambda_{2})=\overset{K_{0}}{\underset{k=1}{\sum }}\beta_{k}\sin\left(\frac{k\pi }{l}(\lambda_{1}-a)+\omega_{k}\right)\sin\left(\frac{k\pi }{l}(\lambda_{2}-a)+\omega_{k}\right),$$ (30)

where

$$\left\{ \begin{array}{l} \omega_{k}=\tan^{-1}\left(\frac{\int_{a}^{b}\int_{a}^{b}C_{0}(\lambda_{1},\lambda_{2})\;\sin\;\frac{k\pi }{l}(\lambda_{1}-a)\;\cos\;\frac{k\pi }{l}(\lambda_{2}-a)\;d\lambda_{1}d\lambda_{2}}{\int_{a}^{b}\int_{a}^{b}C_{0}(\lambda_{1},\lambda_{2})\;\sin\frac{k\pi }{l}(\lambda_{1}-a)\;\sin\frac{k\pi }{l}(\lambda_{2}-a)\;d\lambda_{1}d\lambda_{2}}\right)\\ \beta_{k}=\frac{4}{l^{2}}\int_{a}^{b}\int_{a}^{b}C_{0}(\lambda_{1},\lambda_{2})\;\sin(\frac{k\pi }{l}(\lambda_{1}-a)+\omega_{k})\;\sin\left(\frac{k\pi }{l}(\lambda_{2}-a)+\omega_{k}\right)\;d\lambda_{1}d\lambda_{2} \end{array}\right..$$ (31)

### Distance functions

The distance function or the non-dimensional distance function is critical for formation of the localization matrices $\rho_{{}^{m\times m}}^{non-periodic}$ and $\rho_{{}^{m\times m}}^{periodic}$ in 1D cases based on Gaspari and Cohn [28]. If a 1D model has equal spacing grids, as supposed in the aforesaid 1D cases under periodic and non-periodic boundary conditions, the non-dimensional distances can be expressed as functions with respect to the grid number (*i*). For example, in the 1D periodic case, the non-dimensional distance between two model grid-points can be formulated as *r*_*i*,*j*_ = *d*_*i*,*j*_/*d*_0_ = min(|*i*−*j*|, *m*−1−|*i*−*j*|)/*i*_0_, according to the expressions *λ*_*i*_ = (*i*−1)×*dλ*, *λ*_*j*_ = (*j*−1)×*dλ* and 2*π* = (*m*−1)×*dλ*, where *i*_0_ = *d*_0_/*dλ*. Similarly, the non-dimensional distance between two model grid-points in the 1D non-periodic case can be expressed as *r*_*i*,*j*_ = *d*_*i*,*j*_/*d*_0_ = |*i*−*j*|/*i*_0_, where *i*_0_ = *d*_0_/*dx*. For the non-dimensional distances between a model grid-point and an observation location, the subscript j may be a real number: *j* = 1 + *λ*_*j*_/*dλ*, where *λ*_j_ is the observation location.

In 2D cases, the non-dimensional distance between two model grid-points can also be expressed as functions of grid number (*i*, *j*). If the domain is rectangular with *m*×*n* discrete grids, i.e., [*a*, *b*; *c*, *d*] with the grid-sizes *dx* = (*b*−*a*)/(*m*−1) and *dy* = (*d*−*c*)/(*n*−1), respectively, the distance between any two discrete points A($x_{i_{A}},y_{j_{A}}$) and B($x_{i_{B}},y_{j_{B}}$) in this domain can be defined as $d_{A,B}=\sqrt{{\left(x_{i_{A}}-x_{i_{B}}\right)}^{2}+{\left(y_{j_{A}}-y_{j_{B}}\right)}^{2}}=\sqrt{{\left(i_{A}-i_{B}\right)}^{2}dx^{2}+{\left(j_{A}-j_{B}\right)}^{2}dy^{2}}$, where *x*_*i*_ = *a*+(*i*−1)×*dx* and *y*_*i*_ = *c*+(*j*−1)×*dy*. The corresponding non-dimensional distance is then expressed as $r_{A,B}=d_{A,B}/d_{0}=\sqrt{r_{x}^{2}+r_{y}^{2}}$, where *d*_0_ is the filtering radius, *r*_*x*_ = |*i*_*A*_−*i*_*B*_|/*i*_0_, *r*_*y*_ = |*j*_*A*_−*j*_*B*_|/*j*_0_, *i*_0_ = *d*_0_/*dx* and *j*_0_ = *d*_0_/*dy*. Because the correlation function *C*_0_(*r*) has a close relationship with the exponential function [35]:

$$C_{0}(r_{A,B})∼e^{-\alpha r_{A,B}^{2}}=(e^{-\alpha r_{x}^{2}})(e^{-\alpha r_{y}^{2}}),$$ (32)

where the constant *α* > 0, the correlation function is approximately separable in 2D cases:

$$C_{0}(r_{A,B})\approx C_{0}(r_{x})\cdot C_{0}(r_{y}).$$ (33)

Therefore, we assume that the 2D expansion ${\tilde{C}}_{K_{0}}(r)$ is approximately separable:

$${\tilde{C}}_{K_{0}}(r_{A,B})\approx {\tilde{C}}_{K_{0}}(r_{x})\cdot {\tilde{C}}_{K_{0}}(r_{y}).$$ (34)

It suggests that a 2D correlation function can be calculated using two 1D correlation function, which greatly reduces its complexity in calculations. If (*i*, *j*) is used to express an observation location, *i* and *j* may not be integer numbers, as in the 1D case.

If the 2D domain is the spherical surface with longitude-latitude coordinates: (*λ*, *θ*)∈[0, 2*π*; −*π*/2, *π*/2], which is widely used in global atmospheric models, the exact distance between two discrete points A($\lambda_{i_{A}},\theta_{j_{A}}$) and B($\lambda_{i_{B}},\theta_{j_{B}}$) in this domain is defined as $R_{0}\cos^{-1}(\sin\theta_{j_{A}}\sin\theta_{j_{B}}+\cos\theta_{j_{A}}\cos\theta_{j_{B}}\cos(\lambda_{i_{A}}-\lambda_{i_{A}}))$. However, this formula of distance may lead to inseparability in calculation of the corresponding correlation function. Due to this reason, the distance function here is approximately defined as the hypotenuse of the curved-edge right triangle consisting of the points A, B and O, where the point O can be O_*A*_($\lambda_{i_{B}},\theta_{j_{A}}$) or O_*B*_($\lambda_{i_{A}},\theta_{j_{B}}$). It means two right triangles Δ*BAO*_*A*_ and Δ*ABO*_*B*_ share the same hypotenuse. These two triangles have the same meridional leg length but different lengths of zonal leg ($\bar{AO_{A}}$ and $\bar{O_{B}B}$), which are $d_{\lambda }^{A}=R_{0}\cos\theta_{j_{A}}\times \min(\left|\lambda_{i_{A}}-\lambda_{i_{B}}\right|,2\pi -\left|\lambda_{i_{A}}-\lambda_{i_{B}}\right|)$ and $d_{\lambda }^{B}=R_{0}\cos\theta_{j_{B}}\times \min(\left|\lambda_{i_{A}}-\lambda_{i_{B}}\right|,2\pi -\left|\lambda_{i_{A}}-\lambda_{i_{B}}\right|)$, respectively. The value of the exact distance *d*_*A*,*B*_ is between the hypotenuse lengths of Δ*BAO*_*A*_ and Δ*ABO*_*B*_ that are respectively $d_{A,B}^{A}=\sqrt{{\left(d_{\lambda }^{A}\right)}^{2}+d_{\theta }^{2}}$ and $d_{A,B}^{B}=\sqrt{{\left(d_{\lambda }^{B}\right)}^{2}+d_{\theta }^{2}}$ (where $d_{\theta }=R_{0}\left|\theta_{j_{A}}-\theta_{j_{B}}\right|$), i.e., $\min(d_{A,B}^{A},d_{A,B}^{B})<d_{A,B}<\max(d_{A,B}^{A},d_{A,B}^{B})$. Because the difference between $d_{A,B}^{A}$ and $d_{A,B}^{B}$ is completely due to the difference between $d_{\lambda }^{A}$ and $d_{\lambda }^{B}$ resulted from their different latitudes $\theta_{j_{A}}$ and $\theta_{j_{B}}$, the zonal arc length at the middle of two latitudes $\theta_{M}=(\theta_{j_{A}}+\theta_{j_{B}})/2$, which is $d_{\lambda }=R_{0}\cos\theta_{M}\times \min(\left|\lambda_{i_{A}}-\lambda_{i_{B}}\right|,2\pi -\left|\lambda_{i_{A}}-\lambda_{i_{B}}\right|)$, is used to approximately define the distance between A and B: $d_{A,B}\approx \sqrt{d_{\lambda }^{2}+d_{\theta }^{2}}$. The corresponding non-dimensional distance can similarly be expressed using grid numbers (*i*, *j*): $r_{A,B}=d_{A,B}/d_{0}\approx \sqrt{r_{\lambda }^{2}+r_{\theta }^{2}}$, where *r*_*λ*_ = min(|*i*−*j*|, *m*−1−|*i*−*j*|)/*i*_*θ*_, *r*_*θ*_ = |*j*_*A*_−*j*_*B*_|/*j*_0_, *i*_*θ*_ = *d*_0_/(*R*_0_*dλ* cos *θ*_*M*_) and *j*_0_ = *d*_0_/(*R*_0_*dθ*). In this way, the correlation function for localization in the spherical domain can then be computed using 1D correlation functions according to Eq (34).

To provide an intuitive evaluation on how much the approximation of distance is, we consider a spherical domain with grid-sizes of 4.5° × 4.5°, which is used by the spherical barotropic model in section 3.2. One location is selected at the equator, and the other, at a higher latitude. Table 1 gives the exact arc length between two points on a sphere (the arc length $\bar{AB}$) and the approximate value calculated by $\sqrt{d_{\lambda }^{2}+d_{\theta }^{2}}$. We can see that the longer the distance between two points A and B, the larger the error; and the error at the higher latitude is larger than that near the equator. For example, the error of the distance between point A (90°N, 120°E) and point B (72°N, 138°E) at high latitudes is about 24 km, so the relative error is no more than 2.4%. Compared with the filtering radius used to define non-dimensional distance (e.g., eight grids used by the spherical barotropic model in next section), the influence of such error is much smaller and negligible. The distance errors at lower latitudes are even smaller.

**Table 1:** The exact arc length ($\bar{AB}$) and the approximate value calculated using $\sqrt{d_{\lambda }^{2}+d_{\theta }^{2}}$ between two points A and B on a sphere.

A	B	The arc length $\bar{AB}$	Approximate value
(0°N, 120°E)	(9°N, 129°E)	1412.358	1413.101
(0°N, 120°E)	(18°N, 138°E)	2806.875	2813.190
(90°N, 120°E)	(81°N, 129°E)	1000.754	1003.830
(90°N, 120°E)	(72°N, 138°E)	2001.509	2025.851

### Preliminary evaluation

Given the analytical basis functions shown in Eq (25), numerical tests are conducted to evaluate the expansions of the correlation function with different truncations through comparison with the original one, in the 1D and 2D cases, respectively.

#### 1D case

**Fig 3:**
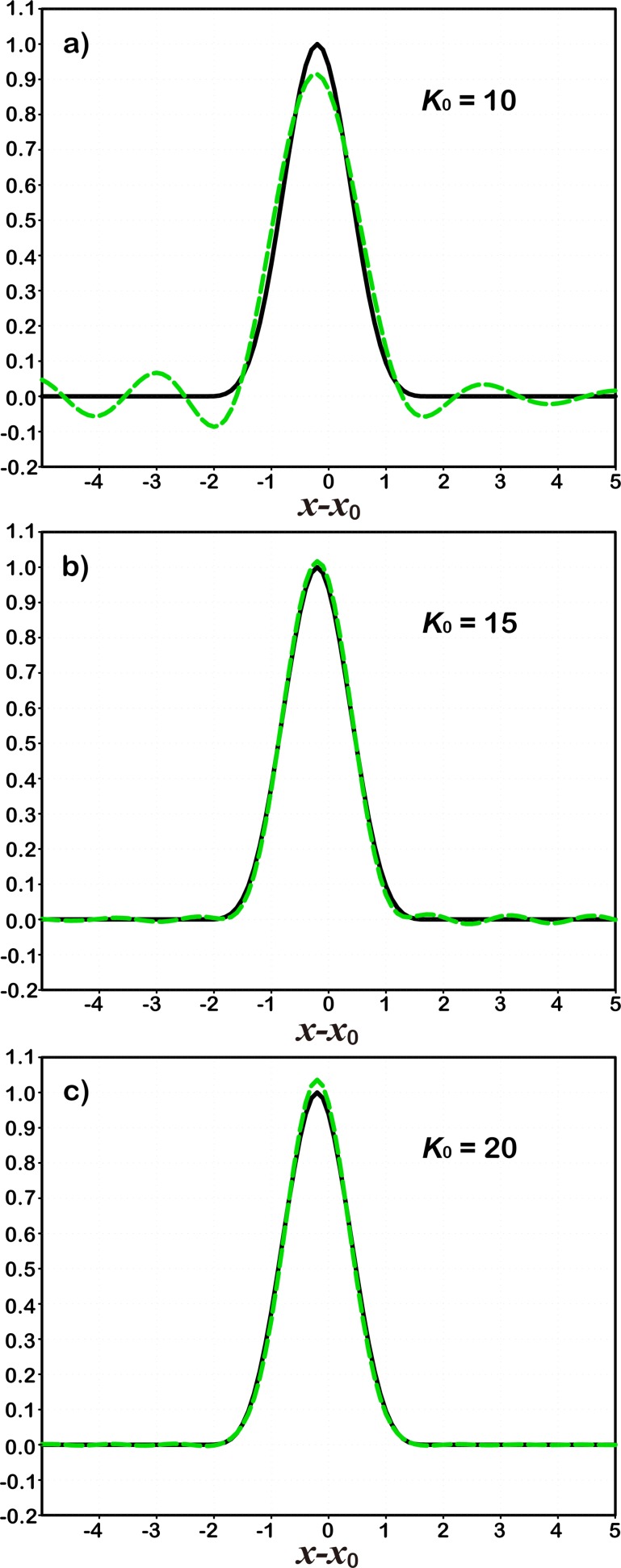
Comparisons between 1D filters presented by the correlation function (black solid) and the expansions (green dashed) with different truncations: *K* = 10 (a), 15 (b), and 20 (c).

As defined in section 2.2, the non-dimensional distance between any *x* ∈ [*a*, *b*] and a prescribed *x*_0_ = ∈ [*a*, *b*] is expressed as *r* = |*x*−*x*_0_|/*d*_0_, where *a* = −5, *b* = 5, and *d*_0_ = 1.0. Setting *x* = *x*_*i*_ (*i* = 1,2,⋯, *m*;*m* = 101) and $x_{0}=x_{i_{0}}$ (*i*_0_ can be any integer number on [1, *m*]; here, we select 49), the original correlation function *C*_0_(*r*_*i*_) (black curves in Fig 3) and its expansion $C_{K_{0}}(x,x_{0})$ (green curves in Fig 3) with different truncation numbers *K*_0_ are then calculated on the discrete grids. It is found that the larger the truncation number *K*_0_ is, the closer to the truth the expansion gets (Fig 3). We can clearly see some fluctuations along the true curve at the location where the correlation coefficients are very small when *K*_0_ = 10 (Fig 3A). As the truncation number increases, these fluctuations become obviously weaker as *K*_0_ = 15 (Fig 3B), and ultimately disappear when using *K*_0_ = 20 (Fig 3C). This means that the first 20 modes form the dominant part of the localization function. In terms of variance contribution, the 20 leading modes account for more than 97% of all modes, no matter how high the resolution becomes (e.g., m = 1001, 10001. see Table 2). In other words, a large number of the remaining modes account for less than 3% of all modes. Table 3 shows that many of the modes have very small eigenvalues, which make very small contributions to the correlation function when fitting observations. Therefore, with a reasonably small number of modes, the new localization will save computational time without sacrificing much accuracy.

**Table 2:** Contributions of accumulated variances of leading modes to the total variance in the 1D case.

Number of leading modes used	Ratio of accumulated variance in the total variance		
	*m* = 101	*m* = 1001	*m* = 10001
1	12.34%	12.14%	12.13%
2	24.29%	28.95%	23.88%
3	35.61%	35.05%	35.02%
4	46.11%	45.41%	45.37%
5	55.62%	54.81%	54.77%
6	64.06%	63.17%	63.12%
7	71.37%	70.43%	70.37%
8	77.55%	76.58%	76.52%
9	82.64%	81.67%	81.61%
10	86.73%	85.78%	85.72%
11	89.94%	89.02%	88.95%
12	92.38%	91.50%	91.43%
13	94.19%	93.35%	93.27%
14	95.50%	94.69%	94.62%
15	96.43%	95.65%	95.57%
16	97.07%	96.32%	96.24%
17	97.51%	96.78%	96.71%
18	97.83%	97.11%	97.04%
19	98.06%	97.35%	97.28%
20	98.24%	97.54%	97.47%
Total variance	1.982459	1.998261	1.999826

**Table 3:** Variances (or eigenvalues) of representative modes in the 1D case.

Mode number	Variance (or eigenvalue)		
	*m* = 101	*m* = 1001	*m* = 10001
1	2.445878E-01	2.425069E-01	2.425069E-01
2	2.368530E-01	2.349722E-01	2.349723E-01
3	2.244613E-01	2.228930E-01	2.228932E-01
4	2.081164E-01	2.069435E-01	2.069438E-01
5	1.887161E-01	1.879857E-01	1.879862E-01
6	1.672772E-01	1.669981E-01	1.669988E-01
7	1.448537E-01	1.449984E-01	1.449992E-01
8	1.224568E-01	1.229669E-01	1.229679E-01
9	1.009850E-01	1.017799E-01	1.017810E-01
10	8.116955E-02	8.215678E-02	8.215796E-02
11	6.354103E-02	6.462652E-02	6.462777E-02
12	4.841677E-02	4.951431E-02	4.951560E-02
13	3.590897E-02	3.694751E-02	3.694882E-02
14	2.595007E-02	2.687822E-02	2.687952E-02
15	1.833037E-02	1.911801E-02	1.911930E-02
16	1.274246E-02	1.337957E-02	1.338084E-02
17	8.826586E-03	9.319923E-03	9.321153E-03
18	6.212055E-03	6.580454E-03	6.581651E-03
19	4.551000E-03	4.820176E-03	4.821343E-03
20	3.542190E-03	3.739829E-03	3.740968E-03
100	9.163717E-06	8.302773E-05	8.402847E-05
1000	/	6.912985E-08	8.006075E-07
10000	/	/	2.463610E-10

**Fig 4:**
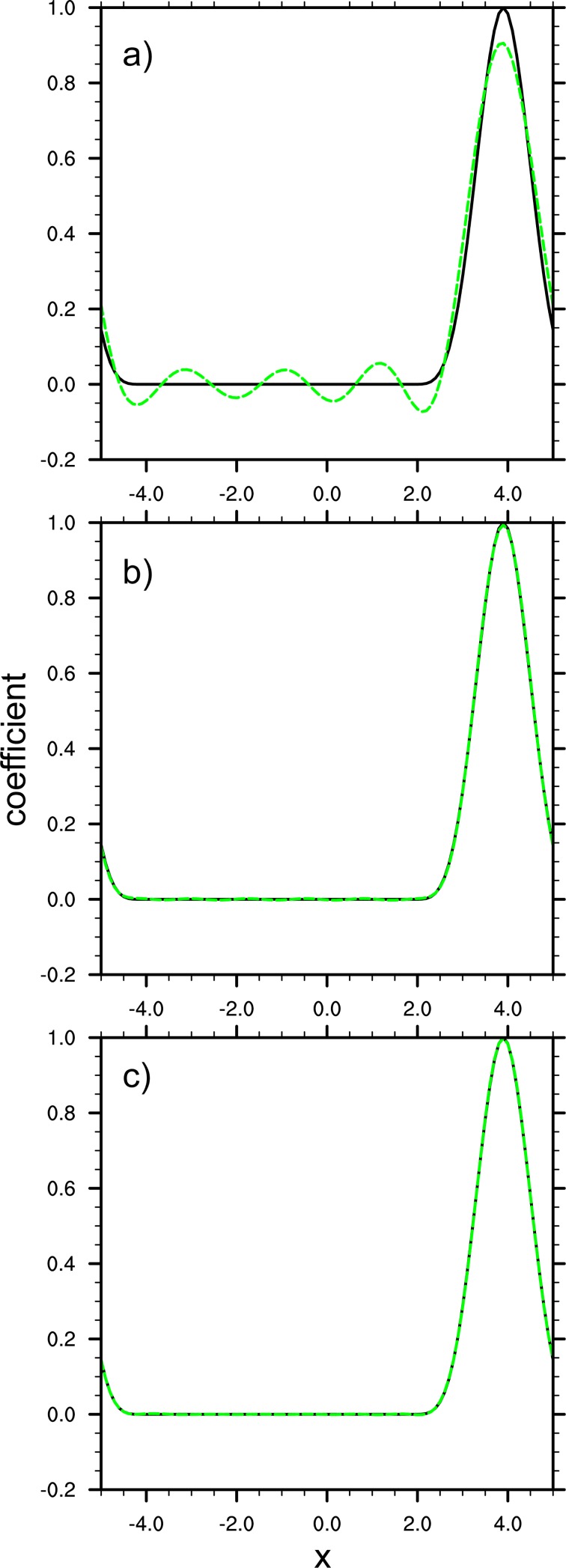
Same as Fig 3, except using a 1D periodic boundary.

Considering a 1D case under periodic boundary condition, with the same experiment setup, but set $x_{0}=x_{i_{0}}$ and select *i*_0_ to be 90. Fig 4 shows the original correlation function *C*_0_(*r*_*i*_) (black curves) and its expansion $C_{K_{0}}(x,x_{0})$ (green curves) with different truncation number *K*_0._ Consistent with our finding in the non-periodic case, increase of the truncation number leads to higher accuracy of the value calculated through our expansion.

#### 2D case

In this case, the non-dimensional distance between two points (*x*, *y*) and (*x*_0_, *y*_0_) is defined as $r=\sqrt{{\left(x-x_{0}\right)}^{2}+{\left(y-y_{0}\right)}^{2}}/d_{0}$, where *x*, *x*_0_ ∈ [*a*, *b*] and *y*, *y*_0_ ∈ [*e*, *f*]. Similar to the 1D case, we set *a* = *e* = −5, *b* = *f* = 5, and *d*_0_ = 1, and uniformly partition [*a*, *b*] and [*e*, *f*] using *m* grids, where *m* = 101. The prescribed point (*x*_0_, *y*_0_) is set to be $x_{0}=x_{i_{0}}$ and $y_{0}=y_{j_{0}}$, and the numbers *i*_0_ and *j*_0_ are selected to be 51.

**Fig 5:**
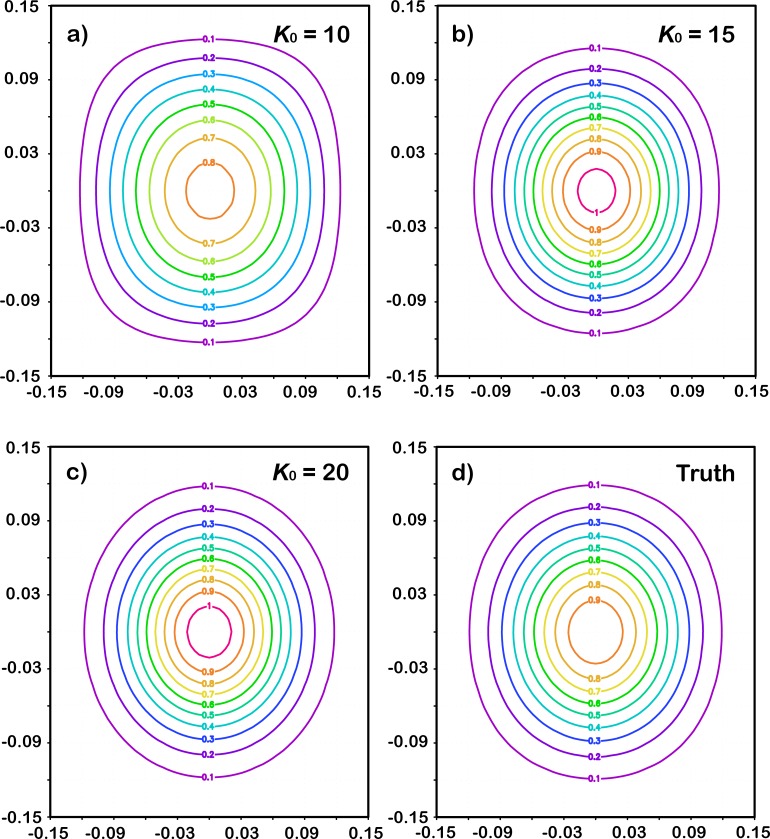
Comparisons between 2D filter presented by the GC correlation function (d) and the expansion with different truncations: *K* = 10 (a); *K* = 15 (b); and *K* = 20 (c).

Fig 5 illustrates a comparison among the 2D filters, separately presented by the expansion ${\tilde{C}}_{K_{0}}(r)$ with different truncations calculated according to Eq (30) (e.g. Fig 5A, Fig 5B and Fig 5C) and the original correlation function defined by Eq (9). The conclusion is similar to that in the 1D case, i.e., the larger the truncation number *K*_0_ gets, the closer to the original correlation function the expansion becomes.

### Assimilation experiments

The above section demonstrates the consistency between the sin basis function and the GC correlation function. Here, we will check it further with some assimilation experiments. An experiment is that assimilating all observations simultaneously in the EnKF with the new localization approach, another way to do, the same as that in some numerical forecast centers, is to assimilate observation serially, one at a time in the EnKF scheme with the GC correlation function. The assimilation experiments are preliminarily tested using observation system simulation experiments (OSSEs) in two models that have increasing complexity: a Lorenz-96 model [36] and a spherical barotropic shallow water model. The “true” state (or “truth”) is defined by a long-term model run, and the corresponding “observations” are generated by adding uncorrelated random noises to the “truth.”

#### Lorenz-96 40-variable model

This model has been widely used to test ensemble-based assimilation methods in a number of earlier studies [14,37]. It is based on the following set of differential equations:

$$\frac{dx_{j}}{dt}=(x_{j+1}-x_{j-2})x_{j-1}-x_{j}+F,$$ (35)

where *j* = 1,2,⋯, *M* is the spatial coordinate; the forcing parameter and the number of spatial elements are set to *F* = 8 and *M* = 40, respectively. The model solves Eq (35) using the fourth-order Runge–Kutta scheme with a time-step of 0.05, where the boundary conditions of Eq (30) are periodic: *x*_*j*+*M*_ = *x*_*j*_ [38]. Simulations during a period of time after a long-term integration (e.g., 10^5^ model time steps) of the model from an arbitrary initial condition are assumed to be the “truth”. Observational data sets include observations of all model variables that are produced by adding uncorrelated random noises with the standard Gaussian distribution (with zero mean and variance of 4.0) to the truth at every step. In this case, the observation number is 40, and no interpolation is needed. The observation error covariance matrix is diagonal. The EnKF is used to assimilate observations at each analysis time step in a cycle with a total of 800 time steps.

We conduct three assimilation experiments: one using 500 members without any localization (named “EXP-1”), and the other two using 20 members with the new (named “EXP-2”) and traditional (named “EXP-3) localization schemes, respectively. The localization radius sets eight grid spacing, and all experiments use the covariance inflation method of Zhang et al.[10]:

$${\left(x_{i}^{'}\right)}_{new}=\alpha {\left(x_{i}^{'}\right)}^{f}+(1-\alpha ){\left(x_{i}^{'}\right)}^{a},$$ (36)

where *α* is the relaxation coefficient; ${\left(x_{i}^{'}\right)}^{a}$ and ${\left(x_{i}^{'}\right)}^{f}$ denote the analysis and the perturbation of the *i*-th ensemble, respectively; and ${\left(x_{i}^{'}\right)}_{new}$ is the final perturbation of the updated ensemble members used for the next assimilation–forecast cycle. In these experiments, *α* = 0.15.

**Fig 6:**
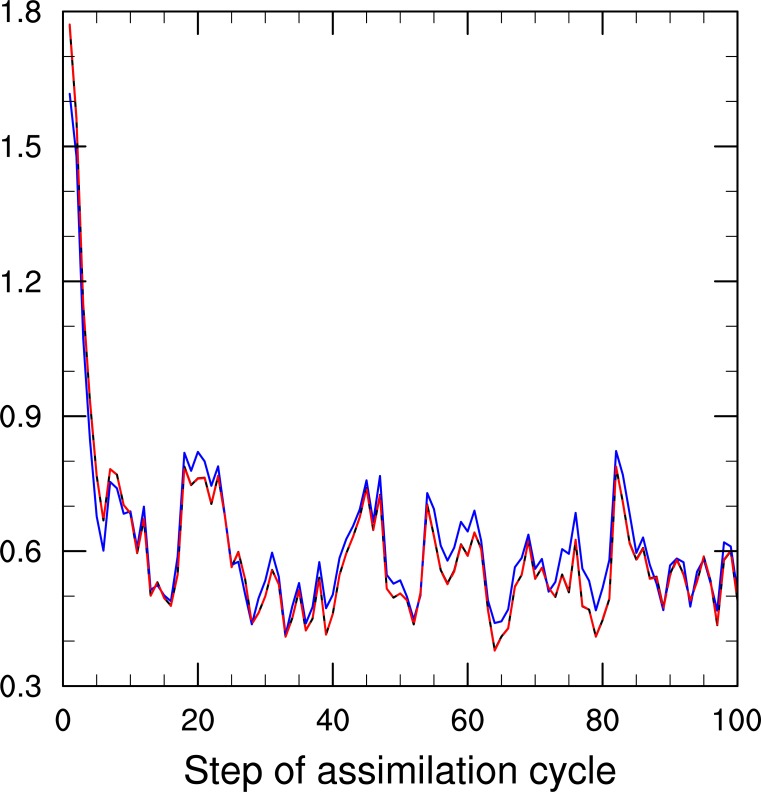
RMSEs of the analysis by the EnKF using 20 samples, respectively, with the new (red line) and traditional (black line) localization schemes and using 500 members without any localization (blue line) during the first 100 steps of the assimilation cycle.

To illustrate the differences among the three experiments more clearly, Fig 6 shows the root-mean-square errors (RMSEs) of the analysis only during the first 100 time steps. The results indicate that the new localization (EXP-2, red line) performs very similarly to the traditional one (EXP-3, black line). The RMSEs of both experiments with localizations are also close to those of the large-size ensemble experiment without the localization (EXP-1, blue line). In terms of overall performances of the three experiments in 800-step assimilation cycles, the new localization (EXP-2) generates the smallest error, of which the time-average RMSE over the 800-step cycles is 0.5558644. The time-averaged RMSEs of the other two experiments are 0.5934035 (EXP-1) and 0.5565553 (EXP-3). Unfortunately, the timesaving nature of the new localization is not obvious in the case of the simple model due to the low dimensions of control variables and observations. Both EXP-2 and EXP-3 used about 20 seconds, which is much more timesaving than the experiment with large ensemble size (EXP-1), which takes 428 seconds in the same computing environment. The significant timesaving characteristics of the new localization scheme become apparent in the following experiments with a more complex model.

#### Spherical barotropic shallow water model

To further compare the performances and computational costs of the new and traditional localizations, we use a spherical barotropic shallow water model to conduct two OSSEs. The model was established using a finite difference scheme with exact energy and mass conservations [39], to solve the following set of equations:

$$\{\begin{array}{l} \frac{\partial u}{\partial t}+\frac{u}{a\cos\theta }\frac{\partial u}{\partial \lambda }+\frac{v}{a}\frac{\partial u}{\partial \theta }+\frac{1}{a\cos\theta }\frac{\partial \varphi }{\partial \lambda }-fv=0\\ \frac{\partial v}{\partial t}+\frac{u}{a\cos\theta }\frac{\partial v}{\partial \lambda }+\frac{v}{a}\frac{\partial v}{\partial \theta }+\frac{1}{a}\frac{\partial \varphi }{\partial \theta }+fu=0\\ \frac{\partial \varphi }{\partial t}+\frac{1}{a\cos\theta }\left[\frac{\partial u\varphi }{\partial \lambda }+\frac{\partial v\cos\theta \varphi }{\partial \theta }\right]=0 \end{array}.$$ (37)

Here, *θ* and *λ* are the latitude and longitude, respectively; *u*, *v* and *φ* denote zonal velocity, meridional velocity and geopotential height, respectively; *a* is the Earth’s radius and *f* is the Coriolis coefficient.

The model has a horizontal resolution of 4.5° × 4.5° (81×41grid points). The initial condition uses the four-wave Rossby–Haurwitz waves. A 20-day integration is conducted first, and the last 10-day integration is taken as the “truth” (i.e., nature run) after a 10-day spin-up. Synthetic observations of geopotential height are created every four gridpoints using the truth plus uncorrelated random noises with the standard Gaussian distribution (with zero mean and variance of 10000.0). In this way, there are 861 *φ* observations in all.

**Fig 7:**
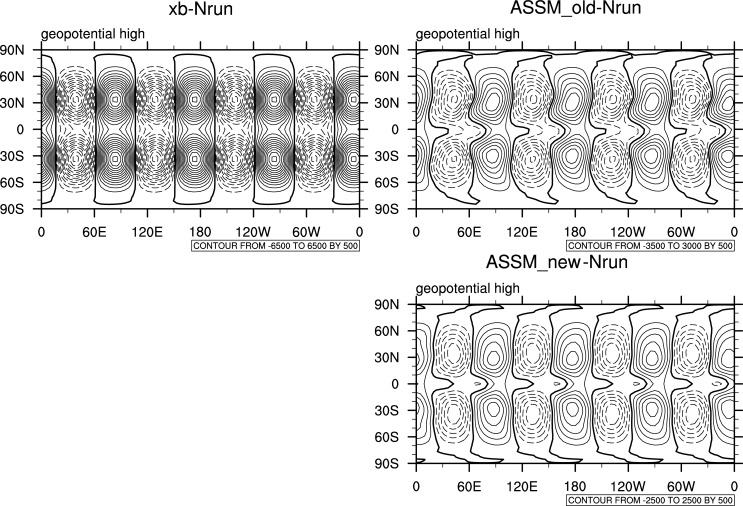
Comparison of the horizontal distributions of geopotential height errors among the background (or first guess), and analyses from ASSM_old and ASSM_new.

A common and easy way to implement the traditional localization in the EnKF is through serial processing [14], which assimilates the observations one by one in a cycle. This is considered to be more timesaving than the traditional localization, but with similar performance. Therefore, the serial implementation method is taken as the traditional localization here, and is compared with the new localization using the spherical barotropic model. Two OSSEs are designed for the comparison: one uses the traditional localization with serial implementation (called “ASSM_old”), and the other adopts the new localization with simultaneous implementation (named “ASSM_new”). The filtering radius in all experiments is eight grid spacing. Fig 7 compares the horizontal error distributions of geopotential height among the background (or first guess), and analyses from ASSM_old and ASSM_new. It shows that all analyses greatly reduce the phase errors of about 30° of longitude existed in the background. In addition, the analysis of ASSM_new at the higher latitude shows marked improvement, compared with ASSM_old. In terms of the RMSE, ASSM_new (1195.982) outperforms the traditional one (1437.468), while all analyses are much better than the background (2752.343). For the computational costs of the two localization schemes, the new localization uses only 25 seconds, far more timesaving than the traditional one, which needs 312 seconds in the same computing environment.

## Discussions

In recent years, ensemble-based approaches have been widely used in various topics, e.g., data assimilation and solutions to conditional nonlinear optimal perturbation [40]. Because an ensemble is generally composed of far fewer members than both the number of observational data and the degrees of freedom of model variables, many spurious correlations between different observation locations, between different model grids, or between observation locations and model grids, occurred. Schür product–based covariance localization has become a practical and powerful tool to make ensemble-based methods perform well even under small ensemble sizes [41]. However, a disadvantage of the traditional localization schemes is their large cost.

When observations assimilated are not many, there is little difference in computational cost between two localization schemes. However, with the large number of observations, even the serial implementation of EnKF, the computational cost is still increased dramatically. Then, if the localization uses a few basis functions to expand, it will be useful for improving work efficiency. This study is a preliminary attempt to develop and improve the localization approach within the EDA process. As the first and necessary step, the new scheme was preliminarily evaluated in its application to a simultaneous assimilation using idealized experiments. Further studies are required in three aspects. First, it is necessary to investigate its role in EDAs for real and complex forecast models. Second, an effort should be made to propose a new serial assimilation scheme in a way of using the leading modes one by one due to the orthogonality between these modes, similar to the way of assimilating the observations one by one in the serial processing of EnKF under the hypothesis of independence between these observations. Third, it is worth exploring how to implement the adaptive localization approach [9,17], because it is now well understood that adaptive localization functions may be more appropriate, although the GC localization function has been widely used in EDA methods. It is anticipated that this work will be challenging due to the noticeable difference between the GC localization function, which is completely independent of ensemble samples, and the adaptive localization functions using complex corrections with respect to ensemble samples. This difference may lead to a great difficulty in expanding the adaptive localization functions using the sine functions as the basis functions, because various eigenvector families may be produced by different ensemble members in the adaptive localization.

## Conclusions

In this paper, we proposed an economical approach to implement covariance localization. We attempted to use a group of basis functions to expand the correlation function, and found that the spatial distributions of the leading eigenvectors of the correlation function are very close to the sine waves that are defined in the domain of definition. We used the sine functions with different frequencies and phases approximately as the basis functions, so that the localization matrix can be decomposed into a series of products of two vectors, and then the Schür product is separable. In this way, the cost of localization can be greatly reduced.

Two numerical tests with different dimensions were conducted to evaluate the expansions of the correlation function. Both tests demonstrated that the larger the truncation number gets, the closer to the original correlation function the expansion becomes. When the truncation number reaches 20, the difference between the expansion and the truth is very small.

The scheme was then verified in an assimilation cycle with the Lorenz-96 model and a single assimilation experiment with a spherical barotropic shallow water model, using OSSEs of the EnKF. In general, when the ensemble size is much larger than the dimension of the model (e.g., 500 for a simple model like the Lorenz-96), the localization has no influence on the assimilation results and is thus not needed. However, if the ensemble size is smaller than the dimension of the model (say, 20), localization is necessary. The experiments conducted using the simple model suggested that ensemble assimilation using a smaller ensemble size with the new localization scheme could achieve a performance comparable to that with the traditional localization, and that applying a large ensemble size without any localization. The new localization even outperformed the traditional one with serial processing in the OSSEs using the spherical barotropic shallow water model. Moreover, the computational cost depends on the number of ensemble members, i.e., the larger the ensemble size gets, the higher the cost becomes. The new localization was shown to be far more timesaving than the serial implementation of the traditional localization in the single assimilation experiments using the spherical barotropic shallow water model, although the timesaving characteristics of the new localization was insignificant in the case of the simple model because of the very low dimension numbers of the control variables and the observations.
